# Case Report: A case of intestinal occlusion caused by endometriosis

**DOI:** 10.3389/fmed.2026.1842868

**Published:** 2026-05-13

**Authors:** Letong Li, Yi Dai

**Affiliations:** 1 Department of Obstetrics and Gynecology, Peking Union Medical College Hospital, Chinese Academy of Medical Science & Peking Union Medical College, Beijing, China; 2 National Clinical Research Center for Women’s Health and Obstetric and Gynecologic Diseases, Beijing, China

**Keywords:** bowel endometriosis, bowel occlusion, case report, endometriosis, multidisciplinary (care or team)

## Abstract

**Background:**

Endometriosis is a common gynecological condition, but intestinal involvement leading to bowel obstruction is rare, occurring in only 0.1–0.7% of cases. Diagnostic delay is frequent due to non-specific gastrointestinal symptoms.

**Case representation:**

A 37-year-old woman presented to the emergency department with abdominal pain and recurrent intestinal obstruction for 10 days. Initial computed tomography (CT) at another hospital showed colonic obstruction, and thus she received gastric intubation, which provided no relief. At our hospital, she underwent emergency exploration surgery, which found suspicious deep infiltrating endometriosis (DIE). After being discharged from the hospital, she attended the outpatient departments of General Surgery and Gynecology alternately, and completed auxiliary examinations. A multidisciplinary team (MDT), including gynecologists, general surgeons, ultrasound specialists, and radiologists, thoroughly and collaboratively assessed the lesion. The gynecologist decided to attempt a diagnostic treatment with gonadotropin-releasing hormone agonist (GnRHa), which finally attributed her symptoms to endometriosis. Subsequent surgery and pathological examination confirmed bowel endometriosis. The patient is now on long-term medical therapy.

**Conclusion:**

This case highlights the diagnostic challenges of bowel endometriosis presenting as intestinal obstruction, and underscores the value of MDT and a diagnostic trial of GnRHa in achieving timely diagnosis and appropriate management.

## 1 Introduction

Endometriosis is a prevalent gynecological disease, affecting approximately 4–17% of menstruating women (1). Endometriosis can be categorized into three types: superficial endometriosis, ovarian endometrioma, and deeply infiltrating endometriosis (DIE). DIE is defined as endometriotic lesions extending more than 5 mm underneath the peritoneum (2).

Intestinal involvement represents a distinct subset, occurring in 3–37% of endometriosis cases (3). Cases of intestinal occlusion due to endometriosis in the small bowel and in the large bowel are even rarer, with a reported prevalence of 0.1–0.7% (4). Diagnostic ambiguity remains a hallmark of intestinal endometriosis.

## 2 Case history

### 2.1 Case description

A 37-year-old woman with a history of chronic constipation (bowel movements occurring every 7–10 days) over the past decade was referred to our emergency department for intestinal occlusion. She reported a history of hysteroscopic myomectomy 3 years ago, after which she suffered from progressive severe dysmenorrhea with a visual analog scale (VAS) score of 10, accompanied by tenesmus and rectal heaviness during menstruation. Approximately 1 year before the current episode, she experienced a 21-day episode of bowel obstruction, which was managed conservatively with lactulose and herbal medicine.

**Figure 1:**
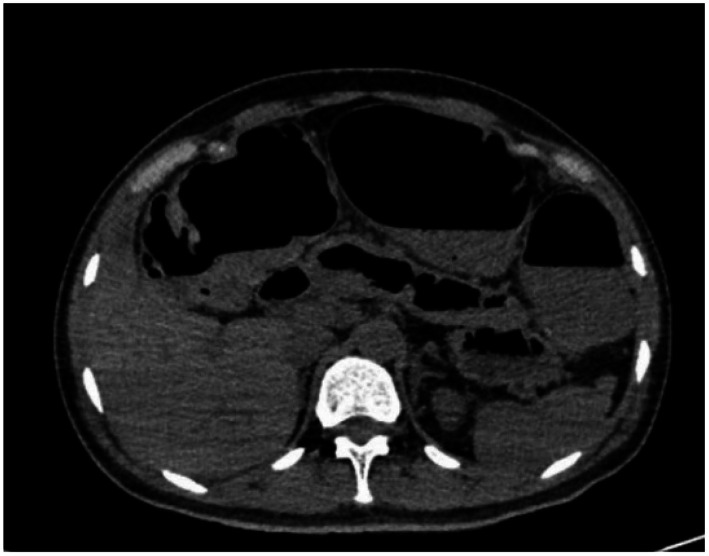
Chest, abdomen, and pelvis CT: multiple small bowel dilations with pneumatosis and multiple fluid levels, consistent with changes in intestinal obstruction.

Ten days before her current admission, abdominal pain and constipation recurred, prompting her to seek care at another hospital. An abdominal CT showed colonic obstruction, and gastric intubation was performed. However, the pain did not ease. She subsequently visited our hospital. Upon arrival, we immediately repeated an abdominal CT scan, which demonstrated a 37 × 30 mm rectosigmoid mass compressing the colon, with diffuse colonic dilation and air-fluid levels (Figure 1). Transvaginal ultrasound confirmed multiple uterine fibroids, the largest one measuring approximately 2.8 × 2.2 cm, located on the posterior wall. Pelvic physical examination identified a fixed, hard extrinsic mass at 7–8 cm from the anal verge, causing complete luminal obstruction.

### 2.2 The first surgery

**Figure 2:**
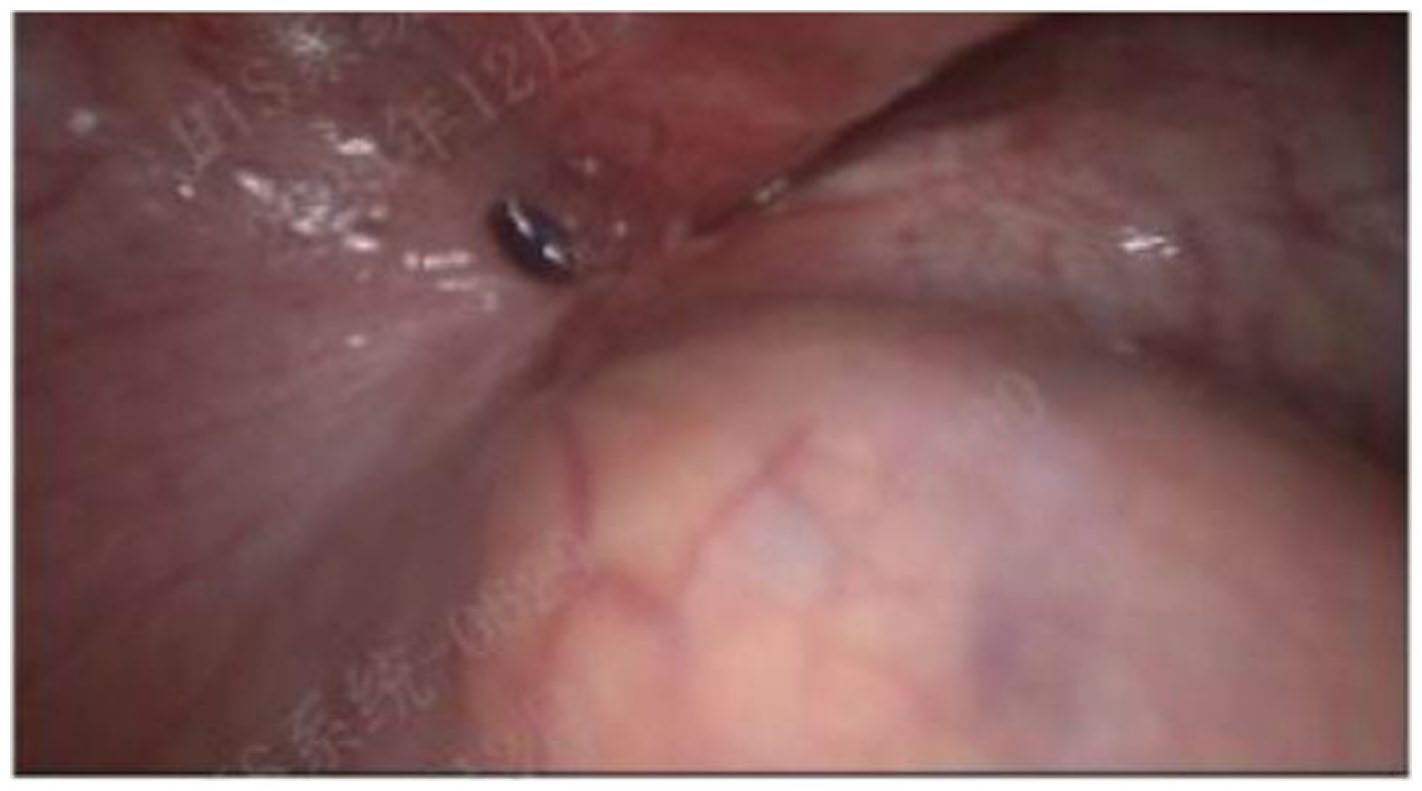
Endometriotic lesion of the Douglas pouch under laparoscopic detection.

**Figure 3:**
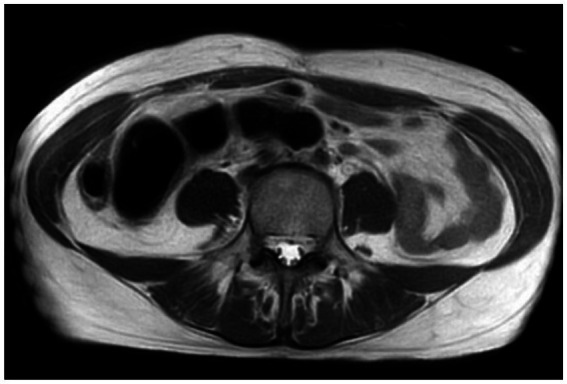
Pelvic MRI: No signs of occlusion.

Both gynecologists and general surgeons suspected that the fibroids were not big enough to cause bowel obstruction. Considering the severe distension, emergency laparoscopic exploration with transverse colostomy was done. During the procedure, the cul-de-sac was difficult to expose, and a single endometriotic lesion was visualized on the serosal surface (Figure 2). Based on intraoperative findings, intestinal obstruction secondary to endometriosis could not be excluded. Five days after the surgery, the patient’s symptoms had resolved. A follow-up pelvic magnetic resonance imaging (MRI) showed colonic distension without signs of obstruction (Figure 3), so the patient was discharged.

### 2.3 Diagnostic odyssey

Later, the patient visited general surgery and gynecology outpatient clinics several times without a definitive diagnosis. Through all the ancillary tests (colonoscopy, CT, transvaginal ultrasound), the origin of the pelvic mass compressing the rectosigmoid remains undetermined.

Three months later, she revisited the endometriosis specialist clinic for further consultation. Physical examination revealed a firm mass in the posterior vaginal fornix, and rectovaginal examination revealed a plate-like, firm nodular mass approximately 5 cm in size with tenderness. Then she underwent endorectal ultrasound, which showed that multiple uterine fibroids remained stable compared with previous imaging. A 2.2 × 2.5 × 0.7 cm plate-like lesion was visualized in the rectovaginal septum and left anterolateral rectal wall, raising suspicion for DIE. Consequently, she received a 3-month trial of GnRHa therapy. Remarkably, after the first dose, the patient regained bowel function, and a follow-up abdominal CT showed a reduction in lesion size. All these responses strongly supported the diagnosis of endometriosis.

### 2.4 The second and third surgery

**Figure 4:**
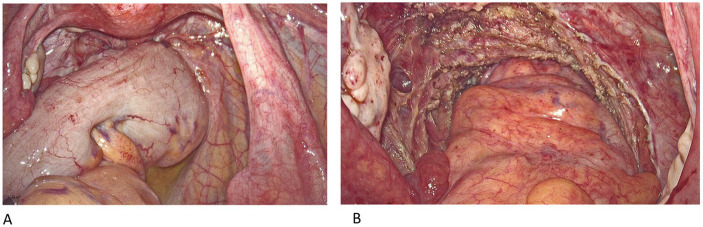
**(A)** The rectouterine pouch is completely obliterated, with adhesion between the anterior rectal wall and the left uterosacral ligament. **(B)** After the surgery.

**Figure 5:**
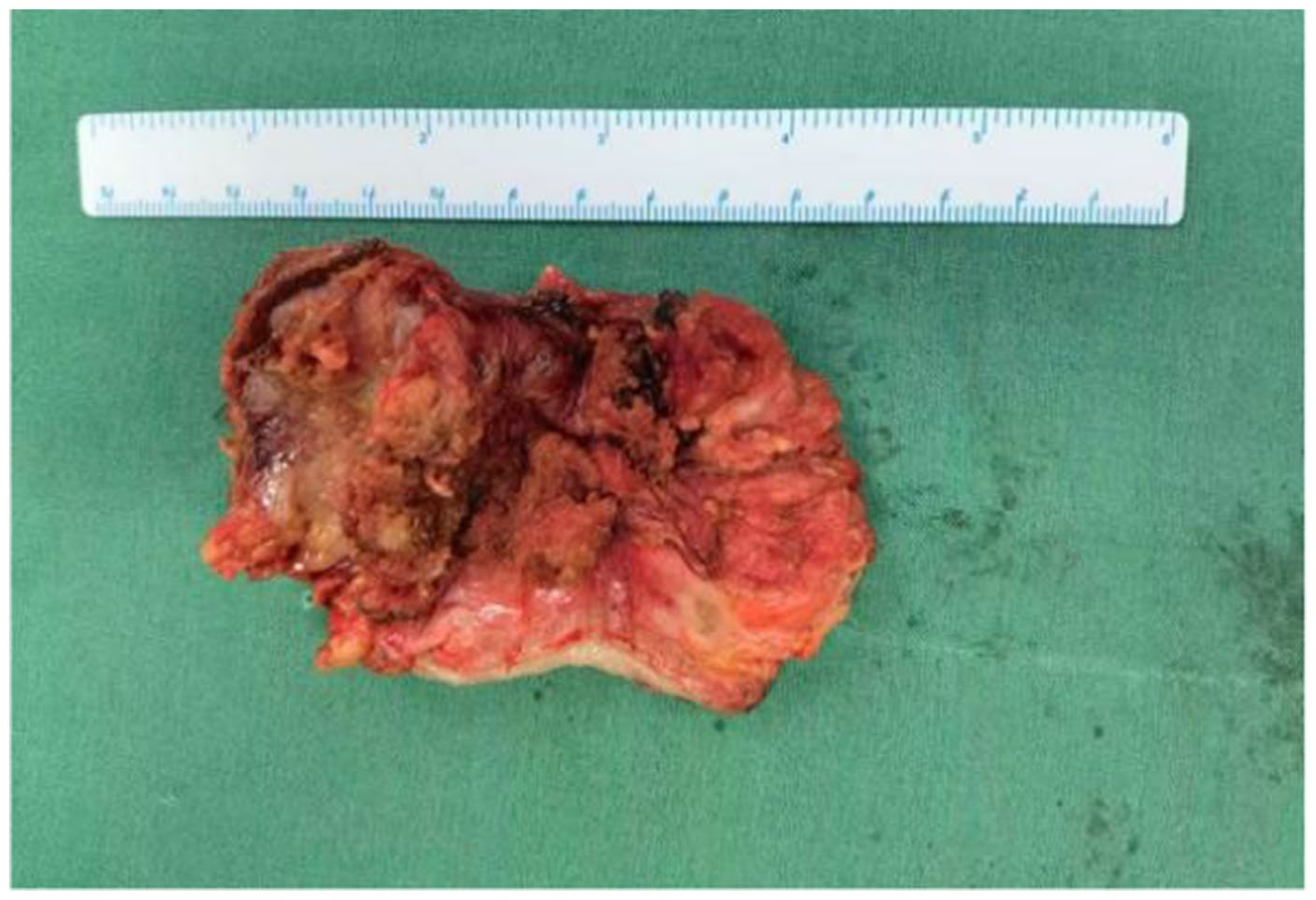
Rectal lesion: The intestinal segment measures 5.5 cm in length and 3.5 cm in circumference.

After a definite diagnosis of endometriosis, she underwent second surgery, which included laparoscopic resection of the rectal endometriotic nodule, uterine myomectomy, and adhesiolysis. The intraoperative findings revealed a completely obliterated left cul-de-sac and dense adhesions between the anterior rectal wall and left uterosacral ligament (Figures 4, 5). The pathologic diagnosis was endometriosis involving the rectal submucosa, muscularis propria, and serosa. No specific findings were noted at both the resection margins of the colon.

Three months after the second surgery, the patient underwent colostomy reversal successfully.

## 3 Outcome and follow-up

**Figure 6:**
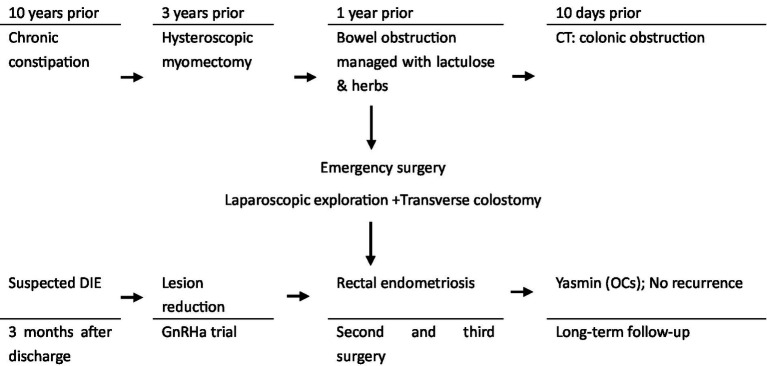
Clinical timeline of the patient’s presentation and management.

After the third surgery, she began long-term management for endometriosis with oral contraceptives (Yasmin). The patient reports satisfactory pain relief and has 1–2 well-formed stools daily without difficulty. A follow-up endorectal ultrasound showed stable uterine fibroids and no evidence of recurrence of endometriosis. Hence, the patient was advised to continue Yasmin and undergo clinical evaluation every 6 months. The whole clinical timeline is summarized in Figure 6.

## 4 Discussion

### 4.1 Diagnostic challenges

Bowel endometriosis is reported to predominate with a prevalence of 3–12% of all endometriosis cases, with the rectosigmoid colon being the most common site (50–90%) (5).

Because many intestinal lesions remain asymptomatic, it is easy to be neglected or misdiagnosed as gastrointestinal disease. Chronic abdominal pain and digestive disorders can also be seen in patients with irritable bowel syndrome (IBS), which affects 15% of the population (6). Some researchers propose that IBS is a manifestation of intestinal endometriosis (7). A diagnostic delay might be caused for approximately 6 to 11 years because of atypical digestive symptoms (8). Our patient had irregular constipation for at least a decade; she went to the doctors several times, but no one connected constipation with endometriosis. Moreover, unlike typical endometriosis cases, our patient did not have ovarian endometriomas, which further obscured the diagnosis during her first surgery. The insight from this case is that early diagnosis and intervention for intestinal endometriotic lesions are essential to prevent disease progression to intestinal obstruction and to better preserve intestinal function. The absence of characteristic endometriotic cysts should not exclude bowel endometriosis, especially in women with cyclic bowel symptoms and a pelvic mass compressing the rectosigmoid.

### 4.2 Role of GnRHa

A key learning point from this case is the successful use of a GnRHa trial to establish the diagnosis. After the first dose, the patient regained bowel function, and follow-up imaging confirmed lesion regression. This response strongly supported endometriosis over malignancy or other bowel diseases. In ambiguous cases where tissue diagnosis is difficult, a short course of GnRHa can serve both diagnostic and therapeutic purposes, potentially avoiding unnecessary bowel resection. Moreover, preoperative GnRHa reduces lesion vascularity and size, facilitating safer surgical resection.

### 4.3 Value of MDT

This case exemplifies the critical role of MDT in managing complex DIE. The team includes gynecologists, general surgeons, urologists, ultrasound specialists, radiologists, and pathologists. In recent years, MDT has been the suggested treatment of bowel endometriosis, which aims at radical removal and improving life quality (9, 10). Our experience highlights that MDT should be implemented early when DIE is suspected, not only for surgical planning but also for diagnostic work-up. The implementation of the MDT is based on three core principles: fertility assessment, organ preservation, and comprehensive pain-psychosocial factors. The collaboration of specialists from different fields enables a comprehensive lesion assessment, appropriate timing of surgery, and an organ-preserving surgical plan.

Comprehensive postoperative management is conducted in order to provide all-around support for the patient’s physical recovery, psychological rehabilitation, and the improvement of quality of life.

Although the MDT model has achieved notable success in practical applications, it still faces challenges and limitations. For example, MDT team members should possess a high level of professional competence. However, due to the current intense medical environment, most experts find it difficult to adhere to the principles of regularly scheduled meetings, designated members, and a fixed venue when participating in case discussions. The fixed MDT committee may encounter difficulties with supervision and implementation. Besides, the follow-up system is imperfect; many outcomes cannot be analyzed quantitatively to evaluate the role of the MDT. These limitations may adversely affect the overall effectiveness and collaborative processes of the MDT.

Nevertheless, with the continuous advancement of medical technology and the in-depth development of interdisciplinary collaboration, the MDT model will be more widely applied and promoted in the treatment of endometriosis. The development of the MDT model still has a long way to go.

### 4.4 Comparison with recent literature

To contextualize our findings within the contemporary literature, we reviewed cases of intestinal obstruction due to endometriosis published in 2025 (Table 1). This overview highlights several important observations. First, diagnostic delay remains a universal challenge; most of the patients had no prior diagnosis of endometriosis before their obstructive episode, and their presenting symptoms frequently mimic other gastrointestinal disorders. Second, MDT involvement of was explicitly noted in several cases, underscoring its growing recognition as a cornerstone of management. Finally, although a diagnostic trial of GnRHa has not been widely reported in other cases, our patient’s favorable response suggests it may be a valuable diagnostic tool in ambiguous presentations.

**Table 1:** Literature review.

	Age	Prior endometriosis history	Symptoms	Imaging findings	Surgical procedure	MDT discussion
Our study	37	No	Chronic constipation, progressive dysmenorrhea, and acute obstruction	CT: Rectosigmoid mass with colonic dilation	Emergency transverse colostomy → laparoscopic rectal nodule resection → colostomy reversal	Yes
Al-Saig et al. (11)	40	No	Lower abdominal pain	CT: Sigmoid colon mass	Emergency Hartmann	Yes
Ro et al. (12)	39	yes	Diagnosed prior. Refractory to hormonal therapy	MRI: Mass formation between the uterus and the rectum	Transverse colostomy → Laparoscopic low anterior resection	Not reported
Attieh et al. (13)	45	No	Nausea, vomiting, abdominal pain, and constipation for 1 week	CT: Terminal ileal lesions Colonoscopy: Obstructed polypoid lesion	Laparoscopic ileo-colic resection	Not reported
Mehrotra et al. (14)	62	No	abdominal pain, nausea, and non-bloody emesis	CT: Small bowel obstruction with a transition point in the right lower quadrant with enteritis	Laparoscopic adhesiolysis and appendectomy	Not reported
Taibi et al. (15)	36	No	Right iliac fossa abdominal pain	CT and MRI: Stenotic lesion in the terminal ileum colonoscopy: normal	Laparoscopic right colectomy	Yes
Boughanmi et al. (16)	37	No	Abdominal pain and vomiting	CT: Distention of the downstream segment	Resection of the invaginated segment and ileo-ileal anastomosis	Not reported
Al Ayoubi et al. (17)	51	No	Abdominal distension, intermittent constipation, and lower abdominal discomfort	Colonoscopy: Non-passable sigmoid stricture	Laparoscopic sigmoidectomy	Not reported
Sumbizi et al. (18)	35	No (But Irregular cycles, menorrhagia)	Abdominal pain, loose stools, and constipation	Colonoscopy: Rectosigmoid polypoid mass, but just inflammatory	Laparoscopic segmental resection and end-to-end stapled anastomosis	Yes
Tahir et al. (19)	37	Yes	Recurrent postprandial vomiting and significant weight loss	CT: 5 cm mass arising from the pylorus and duodenal wall	Whipple	Yes

## 5 Conclusion

Bowel occlusion caused by endometriosis is rare, but it reflects long-term mismanagement. This case demonstrates the value of a structured clinical timeline, a diagnostic GnRHa trial, and multidisciplinary collaboration in achieving a timely diagnosis and optimal surgical outcome. We should always be cautious when a woman of childbearing age complains of intestinal obstruction, while there is no other obvious cause.

## Data Availability

The original contributions presented in the study are included in the article/supplementary material, further inquiries can be directed to the corresponding author.
